# Optimizing Acute Chest Pain Management: Navigating Diagnostic Challenges and Advancing Precision Care

**DOI:** 10.31083/RCM46099

**Published:** 2025-10-29

**Authors:** Meilin Wang, Xiaopu Zhang, Tong Su

**Affiliations:** ^1^Department of Cardiology, The Third Affiliated Hospital of Soochow University, 213000 Changzhou, Jiangsu, China; ^2^Department of Emergency, The Third People's Hospital of Changzhou, 213000 Changzhou, Jiangsu, China; ^3^Department of Cardiology, The First People's Hospital of Changzhou, 213000 Changzhou, Jiangsu, China

Acute chest pain remains a common and diagnostically challenging presentation in the emergency department. Its etiologies range from potentially life-threatening conditions—such as acute coronary syndrome (ACS) and aortic dissection—to less urgent disorders involving the gastrointestinal or musculoskeletal systems. Although guideline targets aim for a missed ACS diagnosis rate below 1%, real-world data suggest rates exceeding 2%, highlighting a persistent diagnostic gap [1]. Concurrently, unnecessary admissions and investigations contribute to increased healthcare costs, emphasizing the importance of refining diagnostic and therapeutic pathways [2, 3, 4]. In this context, the work by Inoue and Minamino, “*How to Approach Patients with Acute Chest Pain*”, provides a systematic synthesis of the essential steps in evaluation and management [5]. By presenting an evidence-based framework, it supports clinicians in addressing a common clinical challenge and helps identify priorities for future research to improve both the accuracy and efficiency in acute chest pain care. The review also briefly notes some of the practical limitations which we discuss, including the risk of bias in algorithmic decision-making and the need for cautious application of accelerated troponin protocols in certain patient subgroups.

The central contribution of this review lies in its emphasis on refining diagnostic precision beyond the traditional “medical history–electrocardiogram (ECG)–biomarker” paradigm. History-taking should focus on the key characteristics of chest pain: radiation to both shoulders or to the right shoulder, which can be indicative of ACS, whereas pain triggered by palpitations or postural changes may help in ruling out ACS, facilitating preliminary risk stratification [6, 7]. Timely and morphologically specific ECG assessment is recommended, with the initial tracing preferably obtained within 10 minutes of presentation and repeat recordings performed within 30–60 minutes as clinically appropriate [8, 9]. Equally critical is the recognition of high-risk patterns such as Wellens’ syndrome and De Winter T-waves, which have been shown to be strongly associated with early identification of acute coronary occlusion, and can meaningfully improve clinical decision-making for ACS, particularly in lesions involving the left anterior descending artery [8, 9, 10, 11, 12].

High-sensitivity cardiac troponin (hs-cTn) testing, when used alongside the “0-hour/1-hour” algorithm, represents an important refinement in the evaluation of acute chest pain [13, 14]. The adoption of assays meeting the International Federation of Clinical Chemistry criteria—defined by a coefficient of variation ≤10% at the 99th percentile and the capacity to detect troponin concentrations in ≥50% of healthy individuals—provides a basis for consistent diagnostic assessment [15]. The structured, quantitative comparison of serial hs-cTn measurements—for example, hs-cTnT <5 ng/L at presentation or <3 ng/L at 1 hour as rule-out thresholds for ACS—represents a significant refinement in assessment. This approach can help reduce subjective variability in clinical decision-making and may support less experienced clinicians in consistently applying protocols, thereby promoting consistency and efficiency across emergency care settings [16]. When such rigorously validated protocols are integrated into routine practice, they have the potential to enhance the speed, precision, and reproducibility of diagnostic evaluation, which are key attributes of high-value cardiovascular care.

The review further illuminates several “gray zones” in acute chest pain management that may challenge conventional algorithms. Approximately 1/3 of patients with non-ST-segment elevation-ACS may present with non-specific or normal ECG findings [17, 18], and in those with hs-cTn below rule-out thresholds, early diagnosis—particularly of unstable angina—remains challenging [7]. This underscores the value of careful risk stratification to guide the identification of patients at potential risk, while helping to limit unnecessary investigations and hospital admissions [3]. In patients with heart failure, elevated hs-cTn levels may obscure concomitant ischemia; in such cases, optimization of heart failure therapy may take precedence. Notably, nearly half (47%) of individuals classified within the rule-out cohort still have underlying coronary atherosclerosis, emphasizing the importance of structured 30-day outpatient followup [2]. Caution is also warranted in the application of risk-scoring systems. The History, ECG, Age, Risk factors and Troponin (HEART) score, developed primarily in low-risk cohorts, may have reduced applicability in higher-risk populations and could potentially misclassify some patients with intermediate risk. Recent validation studies have indicated variability in its specificity and low-risk classification performance across different clinical settings, particularly when applied to populations which differ from those from which it was derived [4, 19]. Likewise, the age weighting incorporated in the Emergency Department Assessment of Chest Pain Score (EDACS) may contribute to the underestimation of risk in younger patients and overestimation in older ones—considerations that warrant adjustment and integration with clinical judgment [19, 20]. Addressing these nuances may be important to help ensure that accelerated diagnostic pathways do not compromise individualized patient care. The review also comments on diagnostic uncertainty in patients with heart failure or renal impairment and notes that accelerated pathways may require clinical judgment to adjust for patient–specific factors.

Building on these considerations, the authors highlight the potential of emerging tools to address some of the limitations of conventional approaches. Machine learning is increasingly being explored as an adjunct to optimize diagnostic and therapeutic strategies [21]. The Collaborative Group for the Diagnosis and Evaluation of ACS (CoDE–ACS) tool, has been reported to reduce the proportion of ACS cases within the observation cohort from 14.6% to 13%, suggesting a modest improvement in triage precision [22]. It should be noted, however, that the integration of such technologies requires careful consideration of potential “heuristic bias”. Clinical reasoning should remain grounded in an initial qualitative assessment—including targeted history-taking and electrocardiographic evaluation—before hs-cTn measurements are applied to refine post-test probability. This sequential approach facilitates the maintenance of a balance between efficiency and accuracy, ensuring that algorithmic support complements rather than replaces clinical judgment.

Future directions in acute chest pain care highlight the transformative potential of artificial intelligence (AI) to enable a paradigm shift from reactive diagnosis to proactive, individualized prevention. This transition is underpinned by the capacity of AI to integrate multimodal data—such as longitudinal electronic health records, advanced imaging, comprehensive biomarker profiles, and continuous wearable-derived metrics–into dynamic, adaptive risk prediction systems.

Accumulating evidence indicates that such multimodal approaches can measurably improve predictive accuracy, with recent studies reporting average gains in the area under the curve of approximately 6% compared with single modality models [23]. Integrating demographic information, biomarker trajectories, and deep learning–derived electrocardiographic features has also been shown to improve short term prediction of major cardiovascular events beyond the performance of conventional strategies [24]. These advances highlight AI’s capacity to deliver real time, patient specific risk assessment and to operationalize precision prevention–shifting the emphasis from acute episode management toward sustained cardiovascular risk mitigation [25, 26] . This represents not merely incremental refinement, but a fundamental redefinition of care pathways.

In summary, this work helps consolidate current approaches in the diagnosis and management of ACS while also addressing an ongoing challenge: aligning standardized protocols with the nuances of individual patient care. Future directions should focus on accelerating the transition from an “experience driven” to a “data driven” paradigm–leveraging robust analytics, validated algorithms, and real world evidence—to achieve the aspirational benchmark of a missed ACS diagnosis rate below 1%. This transition should involve the systematic integration of multimodal data into clinical workflows, prospective validation of risk prediction models across diverse populations, and embedding decision-support tools into electronic health records to enable real-time, individualized guidance. Such developments may contribute to improved patient safety and more judicious use of healthcare resources, supporting both clinical quality and system efficiency.
